# NutriGreen image dataset: a collection of annotated nutrition, organic, and vegan food products

**DOI:** 10.3389/fnut.2024.1342823

**Published:** 2024-03-26

**Authors:** Jan Drole, Igor Pravst, Tome Eftimov, Barbara Koroušić Seljak

**Affiliations:** ^1^Faculty of Computer and Information Science, University of Ljubljana, Ljubljana, Slovenia; ^2^Computer Systems Department, Jožef Stefan Institute, Ljubljana, Slovenia; ^3^Nutrition Institute, Ljubljana, Slovenia; ^4^Biotechnical Faculty, University of Ljubljana, Ljubljana, Slovenia; ^5^VIST–Faculty of Applied Sciences, Ljubljana, Slovenia; ^6^Jožef Stefan International Postgraduate School, Ljubljana, Slovenia

**Keywords:** food label detection, Nutri-Score, BIO, V-label, benchmarking dataset, computer vision

## Abstract

**Introduction:**

In this research, we introduce the NutriGreen dataset, which is a collection of images representing branded food products aimed for training segmentation models for detecting various labels on food packaging. Each image in the dataset comes with three distinct labels: one indicating its nutritional quality using the Nutri-Score, another denoting whether it is vegan or vegetarian origin with the V-label, and a third displaying the EU organic certification (BIO) logo.

**Methods:**

To create the dataset, we have used semi-automatic annotation pipeline that combines domain expert annotation and automatic annotation using a deep learning model.

**Results:**

The dataset comprises a total of 10,472 images. Among these, the Nutri-Score label is distributed across five sub-labels: Nutri-Score grade A with 1,250 images, grade B with 1,107 images, grade C with 867 images, grade D with 1,001 images, and grade E with 967 images. Additionally, there are 870 images featuring the V-Label, 2,328 images showcasing the BIO label, and 3,201 images without before-mentioned labels. Furthermore, we have fine-tuned the YOLOv5 segmentation model to demonstrate the practicality of using these annotated datasets, achieving an impressive accuracy of 94.0%.

**Discussion:**

These promising results indicate that this dataset has significant potential for training innovative systems capable of detecting food labels. Moreover, it can serve as a valuable benchmark dataset for emerging computer vision systems.

## 1 Introduction

It is well established that people’s dietary choices have far-reaching implications, affecting both their health and the environment (1, 2). While food labeling provides a wide selection of information to support consumers in informed food choices, this requires an active role of consumers (3). Assessment of the nutritional value of specific food can be linked to personal needs and health requirements (4). Consideration of nutritional composition in food choices can positively contribute to a person’s overall health - reducing the risk of diet-related diseases such as obesity, diabetes, heart disease, and others (5), while being aware of other food characteristics (such as vegan or organic), aids in promoting sustainability (6). Vegan products are typically free from animal-derived ingredients, reducing the environmental impact associated with livestock farming (7), while organic foods are produced using environmentally friendly farming practices that minimize the use of synthetic chemicals and promote soil health. There are also other relevant aspects of branded foods, for example, designation of origin with geographical indication. It should be noted that consumer food choices are a key driver for changes in the food supply.

When consumers actively seek information about the characteristics of foods and use those in purchasing decisions, this affects both food manufacturers and retailers (8), and also policymakers (9).

**Figure 1:**
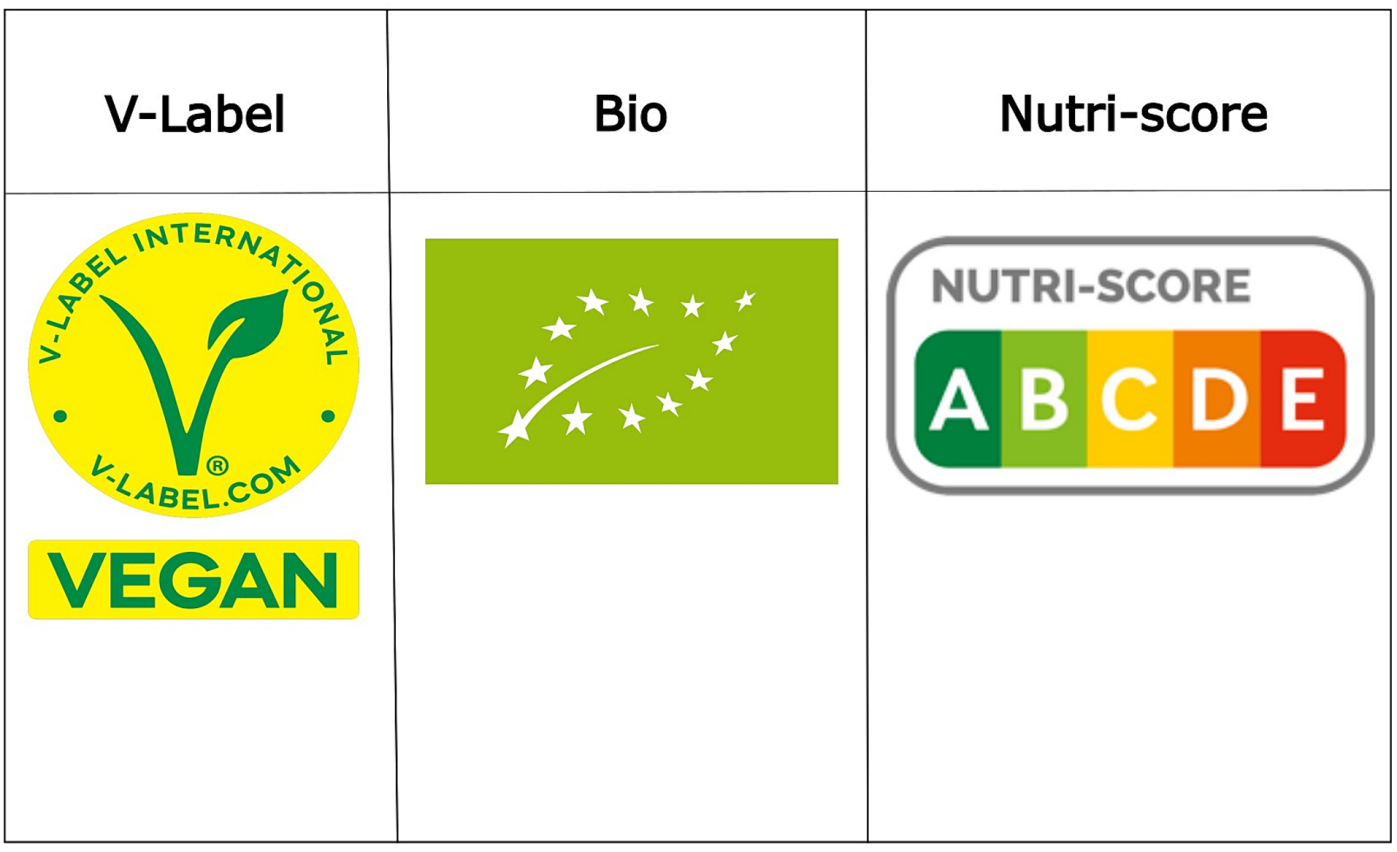
Logos of V-label, BIO, and Nutri-Score labels.

Various labeling standards have been implemented and are displayed on food product packaging to highlight nutritional quality and other food characteristics (10), including the Nutri-Score front of packaging nutrition label (11). This system comprehensively evaluates the overall nutritional quality of food items, allotting grades represented by letters ranging from A (indicating the highest quality) to E (reflecting the lowest quality), accompanied by colors spanning from green to red, respectively. Furthermore, the EU organic logo provides a unified look for EU-produced organic items, aiding consumers in recognizing them and helping farmers’ markets within the EU (12). It’s exclusive to products certified organic by authorized bodies, adhering to rigorous production, processing, and storage standards. The logo is allowed for items with at least 95% organic ingredients, and additional strict criteria for the remaining 5%. The logo is accompanied by a code of the authorized control organization, and origin information for the agricultural raw materials used. Another example is the “V-label,” which is a widely recognized logo that designates products as vegan, reassuring consumers that the item contains no animal-derived ingredients (13). This straightforward label serves as a quick guide for those adhering to a vegan lifestyle, to simplify such ethical and dietary choices. Figure 1 presents the V-label, Bio, and Nutri-Score logos (further referred to with a term label).

The era of digital transformation is bolstered by the capabilities of artificial intelligence (AI) modeling, necessitating a comprehensive food products repository (14). Such repository should encompass not only nutritional data - such as food composition databases (i.e., FCDB) (15) but also other relevant information on food packaging, which are not present in most branded food datasets. Fortunately, emerging technologies involving computer vision as a part of AI, offer assistance in gathering such information for various applications (16, 17).

Computer vision has evolved as a groundbreaking result with remarkable applications in diverse fields, including medical imaging, autonomous vehicles, environmental monitoring, and augmented and virtual reality. Within the realm of food science and nutrition, one of its remarkable applications is food image recognition (16, 17). Food image recognition leverages advanced algorithms and deep learning techniques to interpret visual data, specifically images of food items. At its core, food image recognition involves training deep neural networks using vast datasets of food images. These networks learn to detect and recognize unique features, textures, colors, and shapes associated with different food items. As a result, they can accurately categorize foods into specific classes such as fruits, vegetables, and grains, or even distinguish between different types of dishes. The implications of this technology are far-reaching. For consumers, it promises the convenience of instant nutritional analysis simply by capturing a photo of their meal. This could assist individuals in tracking their dietary choices, making informed decisions, and managing health goals. For researchers and health professionals, it opens up avenues for large-scale dietary assessments, aiding in epidemiological studies and public health interventions.

One example of an already existing model is the NutriNet (16), which is a deep neural network model that can recognize between 520 food and drink items. The model has been trained on a dataset of 130,517 images, from which 54,564 images are food/drink images and 75,953 are other objects unrelated to diet. The dataset has been created by involving 100 images per 520 labels and further post-processed with a series of techniques to create annotated data. Each image contains only a single food that should be recognized. Other food image recognition datasets include UEC-FOOD101 (18), UEC-FOOD256 (19), UEC-FoodPix (20), and UEC-FoodPixComplete (21). The first two datasets contain images with a single food item per image, while the last two datasets include multiple food items per image. The UNIMID2016 dataset (22) also encompasses a multi-food context. Nonetheless, the images were captured within a controlled laboratory environment, where each food item resides on an individual plate, and all plates are positioned on a tray. One of the major challenges of all aforementioned datasets is that the models that are trained on them are not performing well when images from real-life experiences are used. Recently, a new benchmark dataset has been proposed trying to include food images that are coming from real-world life experience (23). The dataset contains images from the MyFoodRepo app (24). It consists of 24,119 food images and a total of 39,325 segmented polygons categorized into 273 different food classes. Even though great effort was made in creating annotated resources that can help the food image recognition challenge, to the best of our knowledge, there is no annotated dataset that consists of food images with standardized labels, referring to nutritional quality and other food characteristics.

Our goal was to develop a large collection of images representing branded food products, which can be used for training segmentation models for detecting various labels on food packaging. The dataset was annotated for the presence of three distinct labels, indicating nutritional quality (Nutri-Score), vegan/vegetarian option (V-label), and organic certification (EU BIO logo). Furthermore, this collection was fine-tuned with YOLOv5 segmentation model to demonstrate the potential of automatic identification of the selected labels. Study results can be used for developing supervised machine-learning models capable of automatically identifying labels in new images with even higher accuracy, eliminating the need for manual assignations.

## 2 Materials and methods

In this section, we briefly describe existing resources utilized for the development of the NutriGreen dataset. Our discussion will be divided into two key components. First, we will delve into the intricate process behind compiling the NutriGreen dataset. Subsequently, we will unveil a pipeline for the annotation of images within this dataset.

### 2.1 Data acquisition

We start by explaining Open Food Facts (25), which provides an API (Application Programming Interface) to collect images of food products, followed by a short introduction of the three labels that are used to annotate the images including Nutri-Score, V-Label, and BIO.

#### 2.1.1 Open Food Facts

**Figure 2:**
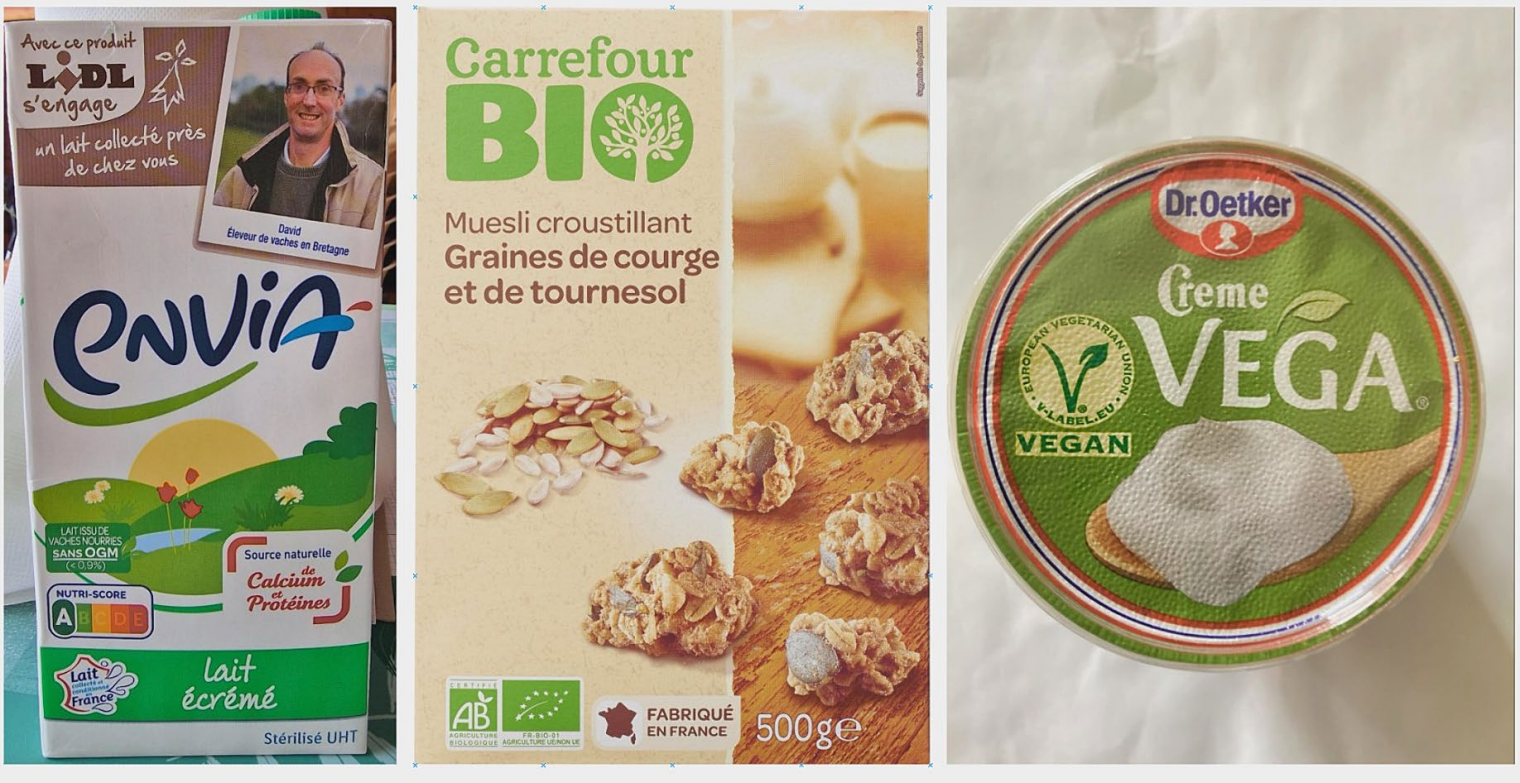
Examples of labeled food product images taken from Open Food Facts.

Open Food Facts (25) is an initiative that leverages open-source principles and crowdsourced data to offer a transparent and accessible database of food product information. It strives to include data from a wide range of countries and regions, contributing to a more comprehensive understanding of food products worldwide. Anyone, whether a nutrition enthusiast, health advocate, or concerned consumer, can contribute data by entering information from product labels or photos of the products. This user-generated data is then validated and processed to improve accuracy and reliability. The platform’s active community of contributors and volunteers helps maintain the accuracy and up-to-date nature of the information. This initiative empowers consumers to make more informed choices about the food they consume by offering a wide range of data, including nutritional content, ingredients, allergens, additives, labels (such as organic or vegan), and more. Open Food Facts offers an API that allows developers to access and integrate its data into various applications, websites, and tools. This fosters the creation of innovative applications that utilize the database’s information to provide users with insights, recommendations, and analysis related to their dietary preferences and health goals. Its impact extends beyond individual consumers, influencing dietary habits, research, policy-making, and the overall landscape of the food industry (Figure 2).

#### 2.1.2 Food labels

##### 2.1.2.1 Nutri-Score

The Nutri-Score label (11), a front-of-pack nutrition labeling system, simplifies complex nutritional information by assigning color-coded and letter-based rankings (A to E) to food products, based on their overall nutritional quality. It considers positive elements like fruits, vegetables, protein, and dietary fiber, as well as negative factors, such as the content of sugars, salt, and saturated fats. This easy-to-understand label aids consumers in making healthier choices and has the potential to drive industry reformulation and influence policy decisions to combat diet-related health issues. Despite very diverse debates about the benefits and challenges of the Nutri-Score, its adoption in multiple countries highlights its potential for promoting nutritional awareness and healthier eating habits.

##### 2.1.2.2 BIO

The European Union organic (BIO) logo food label (12) gives a coherent visual identity to organic products makes it easier for consumers to identify organic products and helps farmers to market them across the entire EU. The organic logo can only be used on products that have been certified as organic by an authorized control agency or body. This means that they have fulfilled strict conditions on how they must be produced, processed, transported, and stored. The logo can only be used on products when they contain at least 95% organic ingredients and additionally, respect further strict conditions for the remaining 5%. The same ingredient cannot be present in organic and non-organic forms. The BIO logo empowers consumers to make ethical and environmentally conscious choices while encouraging food producers to expand their range of organic offerings in response to growing demand, ultimately fostering a more sustainable food system.

##### 2.1.2.3 Vegan

The V-label is an internationally recognized emblem used on food packaging to signify products suitable for vegetarian or vegan diets (13). By prominently featuring this logo, food manufacturers provide clarity and assurance to consumers seeking animal-free options, streamlining decision-making and promoting transparency. Beyond individual choices, the V-label influences industry innovation, encouraging the development of plant-based offerings to meet the growing demand for such products. Its role extends beyond dietary preferences, contributing to a more informed and sustainable food landscape.

### 2.2 NutriGreen annotation pipeline

**Figure 3:**
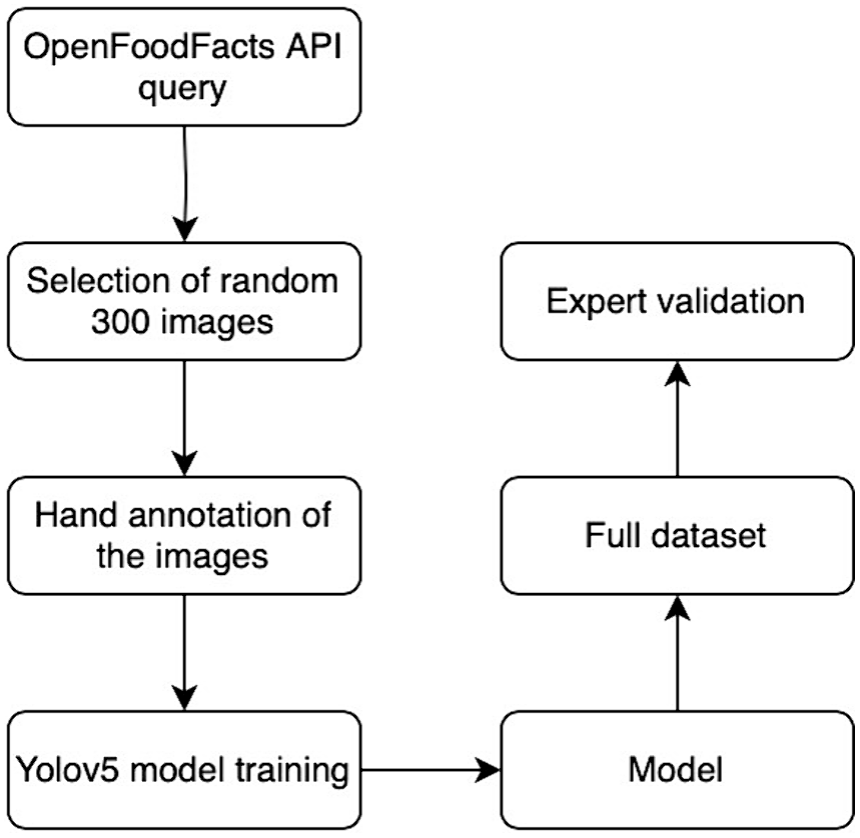
The NutriGreen annotation flowchart.

The NutriGreen annotation pipeline is presented in Figure 3.

## 3 Results

The work has been done in four steps, described in the following sections. To initiate the process, we began by gathering a comprehensive dataset comprising images of branded foods with labels. Subsequently, manual annotations were applied to these images, followed by model training. Continuing the work, we harnessed the power of fine-tuned YOLO models to facilitate automatic annotation. The culmination of our efforts saw a thorough validation of results, a critical step overseen by subject domain experts.

### 3.1 Data collection

To collect the images involved in the NutriGreen dataset, we utilize the Open Food Facts API. The usage of the product images from the Open Food Facts is based on the Creative Commons Attribution ShareAlike license (CC-BY-SA), which allows us to copy and redistribute the material in any medium or format (share), and remix, transform, and build upon the material for any purpose, even commercially (adapt). The collection consists of the following steps:

- Invoking the GET all food products API, which returns information about all food products available in the Open Food Facts database. Here, the output is a big JSON file with information about all products, where each product is represented by its barcode number. This call allows us to collect the barcode numbers, i.e., unique identifiers for all available food products. To refine our search, we employed specific tags such as “labels_tags = vegan,” “labels_tags = bio,” and “page_size = 1,000” in the API request. These tags allowed us to filter the food products based on particular criteria, such as vegan or organic labels. This approach streamlined the process of retrieving product data, ensuring that we obtained only the information relevant to our research and analysis.
- The barcode numbers of the subset of products that result from the filtering are further used to retrieve the images. To retrieve them we have applied two rules based on regular expressions. If the length of the barcode number is less or equal to eight, all images related to that food product can be accessed at a link in the following format: https://openfoodfacts-images.s3.eu-west-3.amazonaws.com/data/{barcode}, where the barcode should be changed with the product name barcode identifier. On the opposite (i.e., if the barcode length exceeds eight characters) the following regular expression (regex) pattern is employed to partition the barcode into subfolders: r‘‘^(..)(..)(..)(.*)$.” For instance, the barcode number 3435660768163 is partitioned as follows: 343/566/076/8163.

The retrieved images are further categorized based on the three different labels related to “Nutri-Score,” “BIO,” and “Vegan.” In addition, the NutriScore label has been split into five different sub-labels such as “Nutri-ScoreA,” “Nutri-ScoreB,” “Nutri-ScoreC,” “Nutri-ScoreD,” and “Nutri-ScoreE.” We need to mention here that retrieving the images using the Open Food Facts can be related to one or more of the labels that are of our interest, however, image segmentation has not been done (collection of regions of pixels that are represented by the label), which is the goal of our study.

### 3.2 Manual annotation and model training

**Figure 4:**
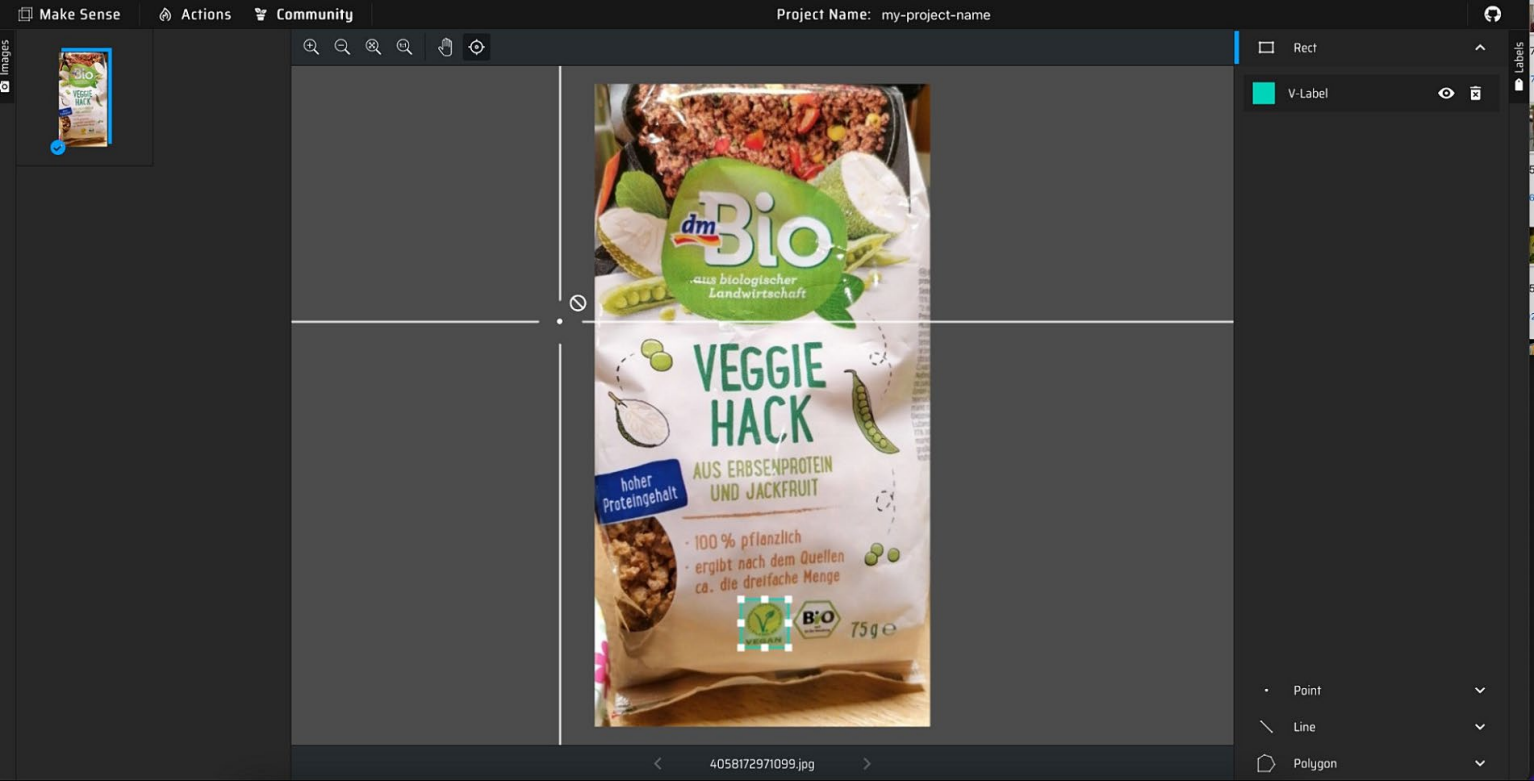
Manual annotation using MakeSense.ai.

To start with image segment annotation, or annotating the collection of regions of pixels that are represented by the label, we randomly select 300 images per label (here we treat each Nutri-Score label as a separate one) for manual annotation. For this task, we have used an open-source tool called MakeSense.ai which allowed us to effortlessly do the job. These are the steps we took (also presented in Figure 4):

- Upload the pictures to the tool and select “Object Detection.”
- Click on “Your label list is empty,” where you add a new class with the name of the symbol you want to annotate eg. “BIO” and start the project.
- Create a selection around the symbol (so that the entire symbol is within the rectangle and there is as little excess as possible). On the right side, select the previously created class that represents your label.
- Go over the selected images by clicking on them and repeat the previous step.
- When finished with annotating, export the selections in YOLO format by clicking the “Actions” button in the top left corner then “Export Annotations” and select “A .zip package containing files in YOLO format.”

After having the ground truth for each label which consists of 300 images per label, we use them as training data for fine-tuning the YOLOv5 model (26). YOLOv5 belongs to the You Only Look Once (YOLO) series of computer vision models, predominantly employed for object detection. It is available in four primary iterations: small (s), medium (m), large (l), and extra-large (x), with increasing levels of accuracy. Additionally, each variant requires varying durations for training. In our study, for each label separately, we fine-tune the YOLOv5 (x) model, so we end up with seven different fine-tuned YOLOv5 (x) models (further we referred to them as YOLO models), which are different in the weights in the deep neural network architecture.

### 3.3 Automatic annotation using the fine-tuned YOLO models

Having the seven fine-tuned YOLO models, we used them for the automatic annotation of the reminder images that were not selected for the manual annotation. Since the images have already been categorized into different labels, for annotating the remaining images per label, the fine-tuned YOLO model for that label is utilized. The collection of annotated images represents a silver standard of NutriGreen since the automated annotated images can still have some false discoveries depending on the performance of the fine-tuned YOLO models, which should be further corrected by expert validation.

### 3.4 Expert’s validation of the automatic annotations

In this step, all automated annotations have gone through another expert validation in order to correct the errors that were produced by the fine-tuned models. With this step, a gold standard for each label has been obtained.

It is also important to note here that some images can encompass multiple labels. For example, a food product can have the “BIO” and “Nutri-ScoreA” labels. To tackle this problem, new seven fine-tuned YOLOv5 (x) models have been developed using the gold standard for each label. Further, these models have been applied to annotate all images, resulting in the automatic generation of all labels for each product. Again, all automated annotations have been further validated and corrected by experts. We need to point out here that the annotations have been checked by two experts from food science. However, the validation does not require any special food science knowledge since it just checks if a particular label (logo) is visible on the image or not. Finally, all images have been collected together forming the NutriGreen gold dataset. Each image contains all labels assigned to it.

## 4 Discussion

**Figure 5:**
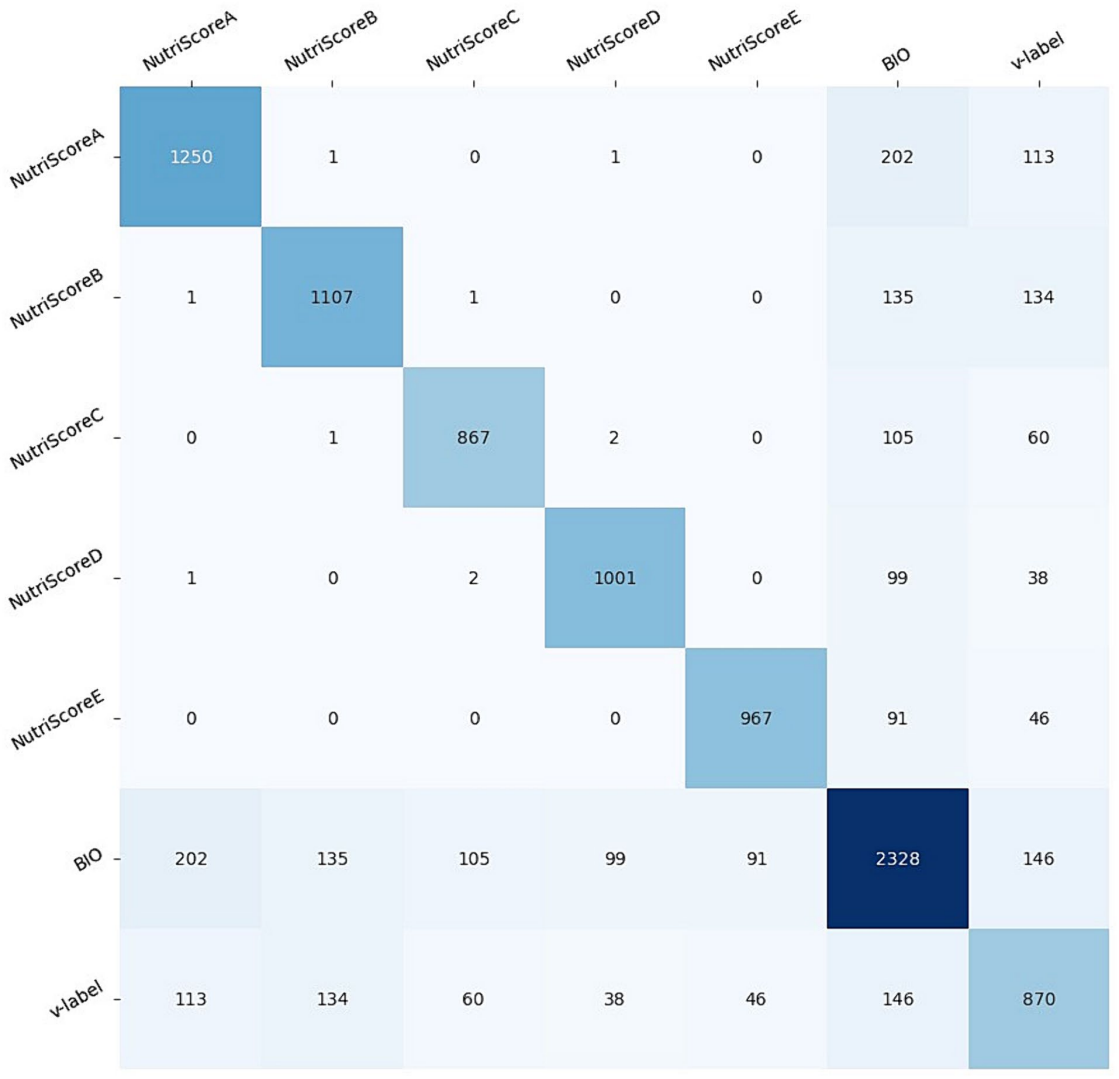
Co-occurrence matrix between the NutriGreen labels.

Performing the NutriGreen annotation pipeline, we have ended up with 2,328 BIO, 870 V-label, 1,250 Nutri-ScoreA, 1,107 Nutri-ScoreB, 867 Nutri-ScoreC, 1,001 Nutri-ScoreD, 967 Nutri-ScoreE images. It can also happen that one image contains more labels; for example, the BIO and V-label might also appear together with one from the Nutri-score label. To show these patterns, Figure 5 presents the co-occurrence matrix between the labels. From the co-occurrence matrix, we can see that the co-occurrence is higher between BIO and V-label with each of NutriScoreA and NutriScoreB than the co-occurrence with NutriScoreC-D-E labels. It follows that many of the BIO and V-label products were classified as more healthy products. In our dataset, there are only 146 images that are classified as both V-label and BIO.

**Figure 6:**
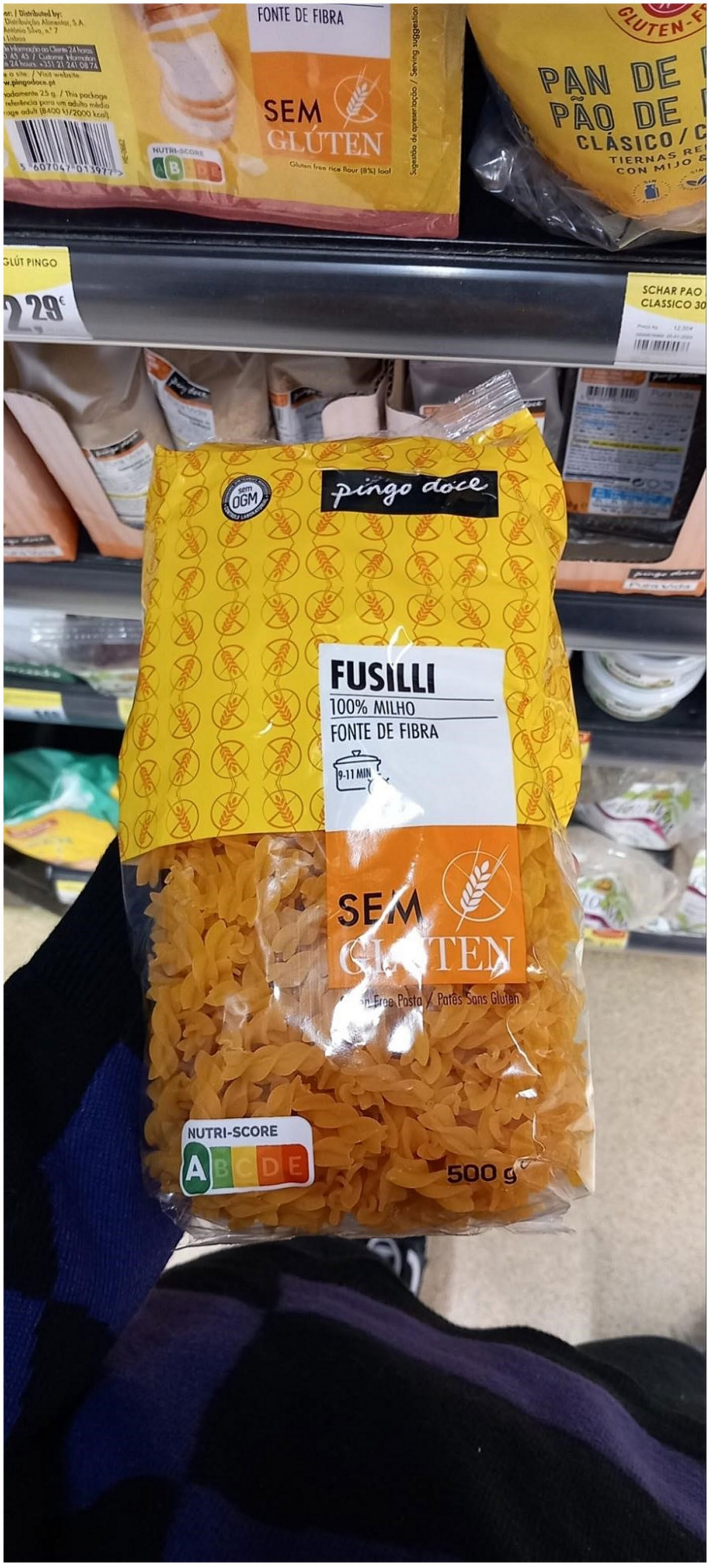
An example of an image with Nutri-ScoreA and Nutri-ScoreB.

**Figure 7:**
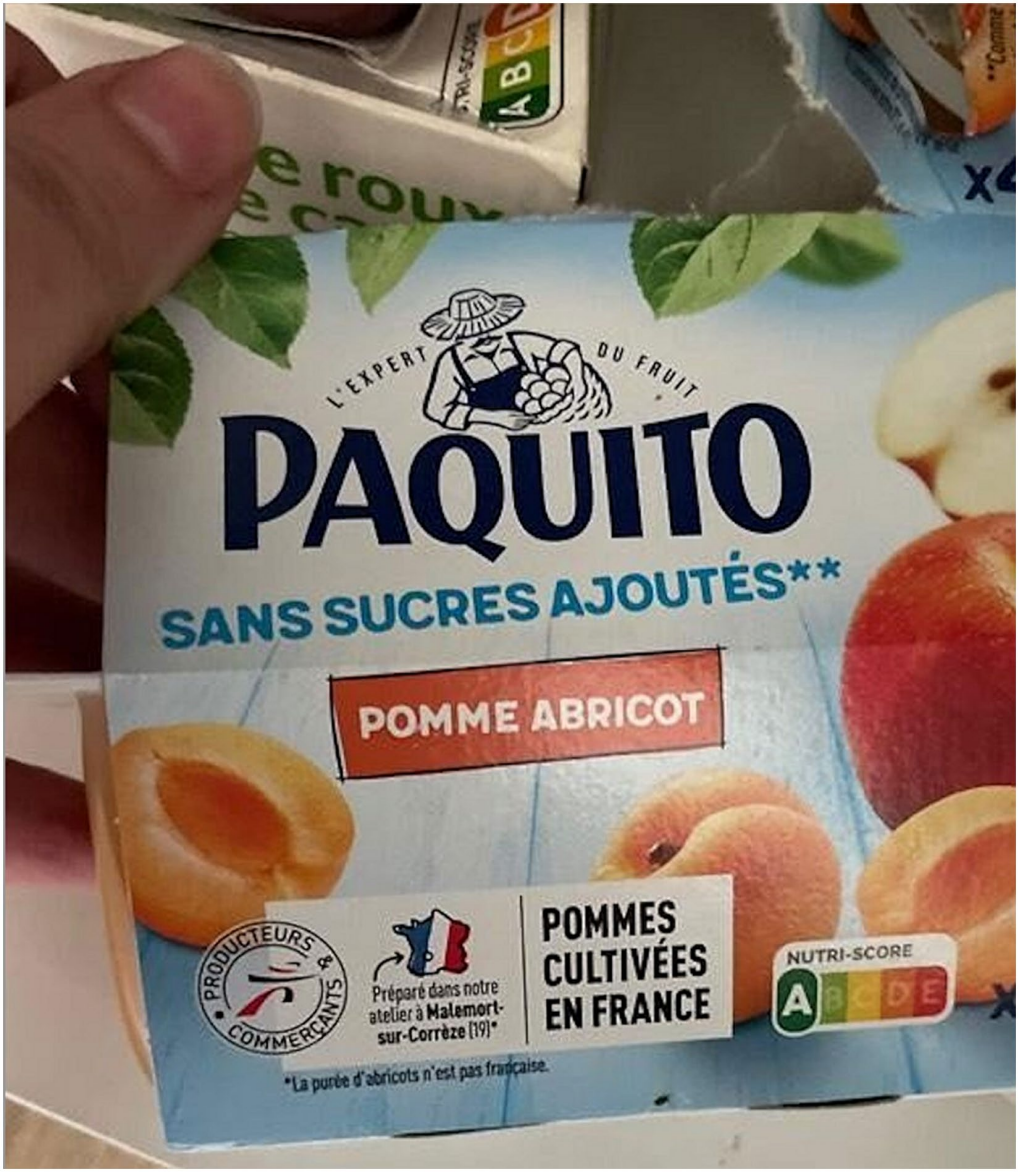
An example of an image with Nutri-ScoreA and Nutri-ScoreD.

**Figure 8:**
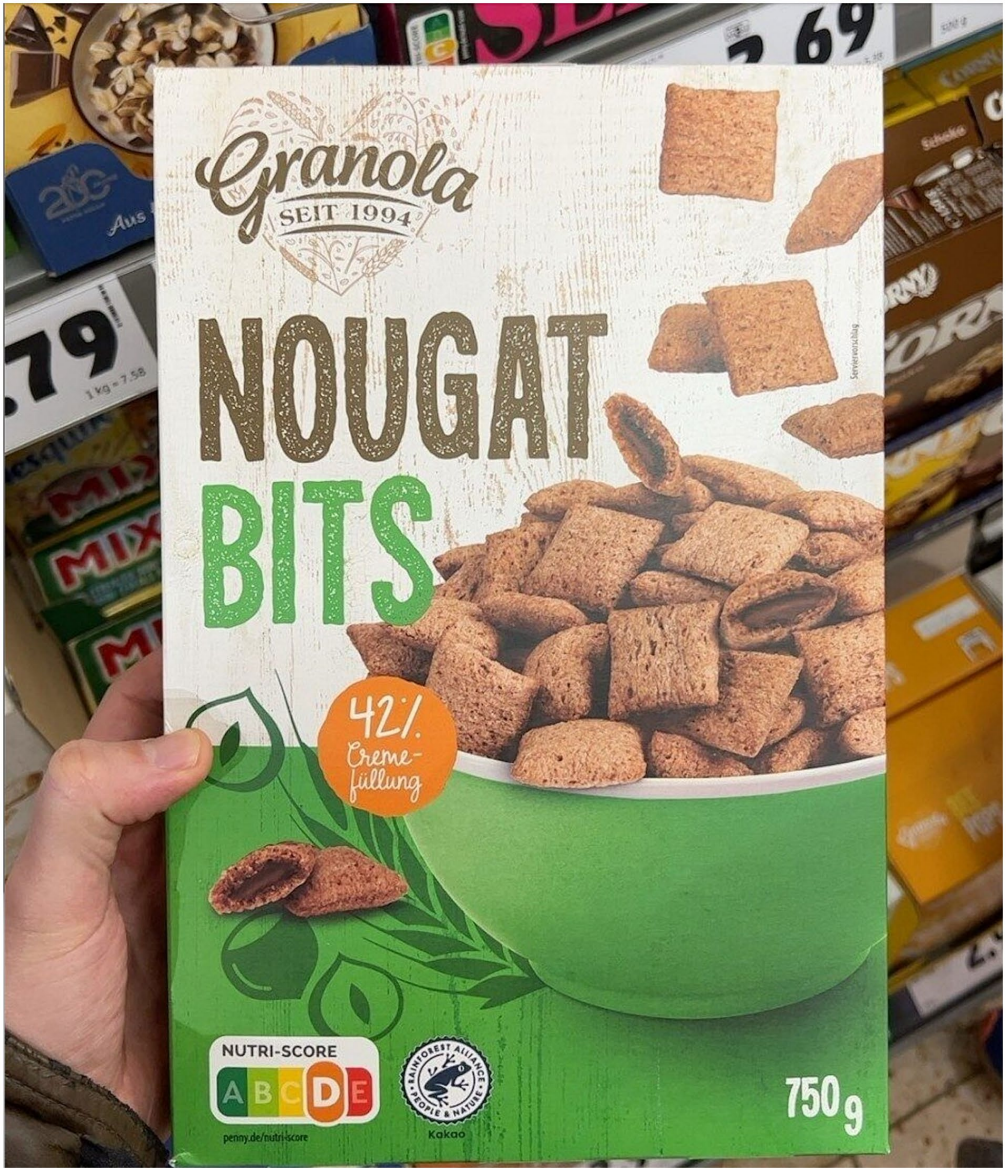
An example of an image with Nutri-ScoreC and Nutri-ScoreD.

By using the co-occurrence matrix, we can detect that there is one image for which there is an annotation NutriScoreA and NutriScoreB, one that contains NutriScoreA and NutriScoreD, and two images containing NutriScoreC and NutriScoreD. Since each product can have only a single Nutril-Score, these results point out that we need to make some further investigations. Figures 6–8 present an example of the images with more NutriScore labels respectively: (i) NutriScore-A and NutriScore-B, (ii) NutriScore-A and NutriScore-D, and (iii) NutriScore-C and NutriScore-D. Looking at those examples we can see that there is a primary label that is actually presented on the image of the food product, however, there is also a secondary NutriScore label that is detected in the background of the image on another food item. Those annotations originate from the automatic annotation step, where we labeled the images using the YOLOv5 model. We need to point out here that going through the expert validation, we did not correct those labels since they are both presented on the image.

**Figure 9:**
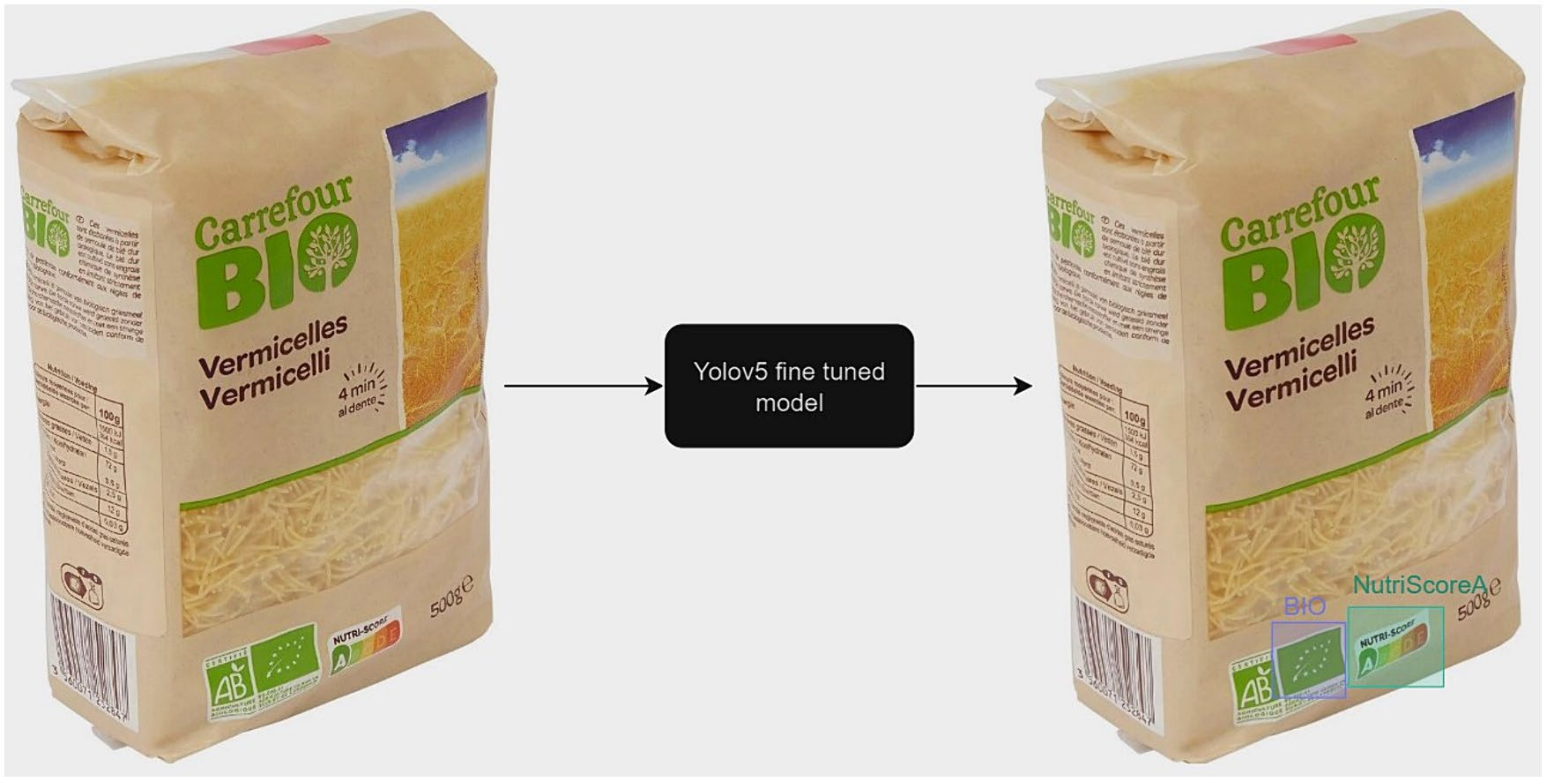
The workflow of the YOLOv5 model fine-tuned on the NutriGreen dataset.

**Figure 10:**
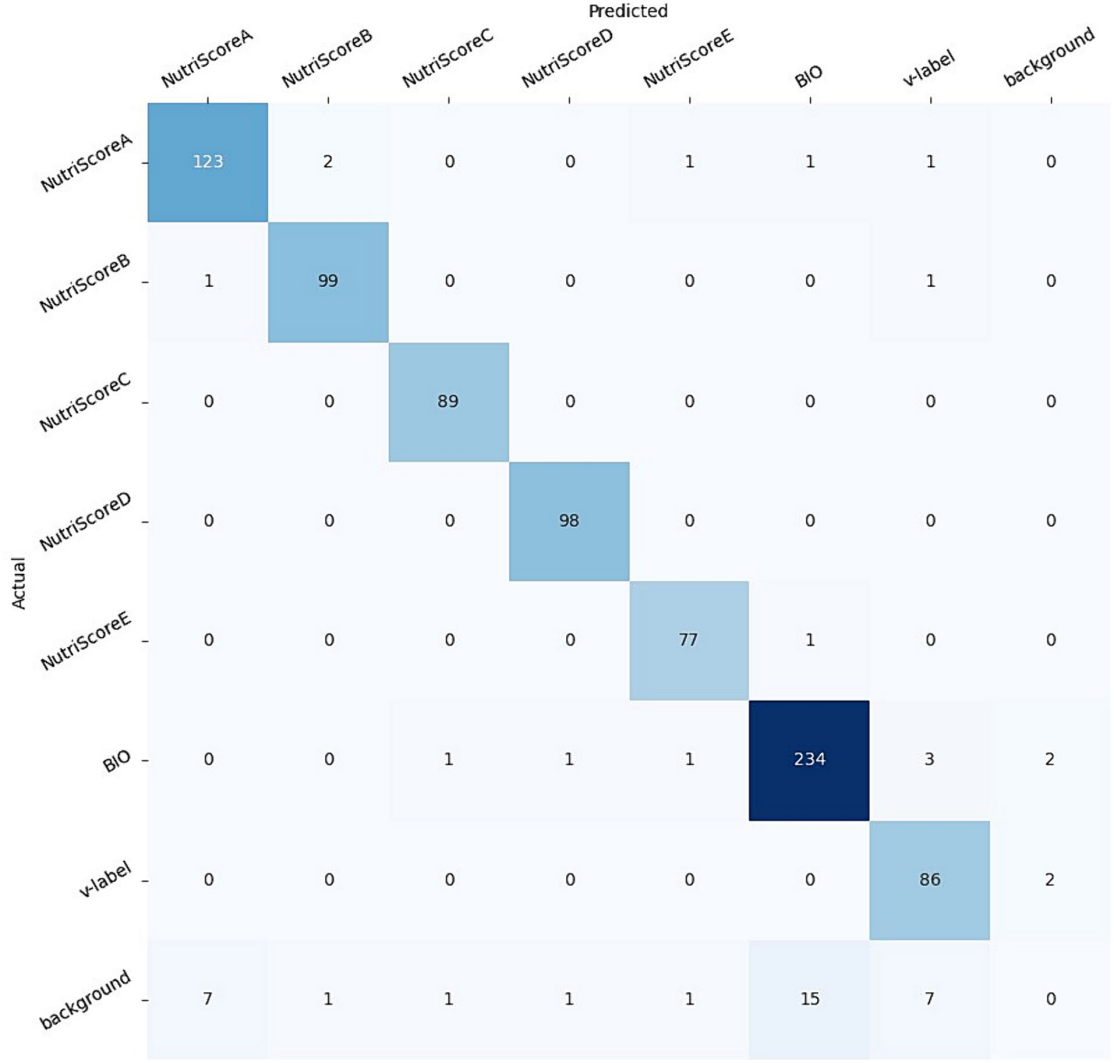
The confusion matrix of the YOLOv5 model fine-tuned on the NutriGreen dataset.

Next, to show the utility of the NutriGreen dataset, we have used it to train a YOLOv5 model that is able to detect the labels. The workflow of the model is presented in Figure 9. For this purpose, we have split the dataset into train (70%), validation (20%) and test (10%). The split has been stratified, which means that we used 70% of the images from each label in the train set, 20% in the validation, and 10% of each label in the test split. The results of the evaluation led to an accuracy of 94% on the test set and 93.9% on the validation set. The confusion matrix is presented in Figure 10. We need to clarify here that our goal is not to find the best model, but we would like to show the utility of the dataset and that it consists of powerful information for performing modeling. This has been the reason why we have not made several splits to show the robustness of the model and with this, we save a lot of computational power and energy.

The confusion matrix has shown that the model is accurate for all labels (five Nutri-Score, the Bio, and the V-Label), with a small percentage of each label that is not detected and located: 4% of the NutriScore-A, 1% of the NutriScore-B, 1% of the NutriScore-E, 3% of the BIO, and 2% of the V-label. The NutriScore-C and D have been detected with 100% accuracy. We need to point out here that these results can change with different splits, however, we do not expect bigger deviations in the accuracies obtained.

## 5 Conclusion

In this study, we present the NutriGreen dataset, a compilation of images portraying branded food products. The dataset is designed for training segmentation models to identify various labels on food packaging. Each image within this dataset features three distinct labels: one that assesses its nutritional quality through the Nutri-Score, another that designates its vegan or vegetarian origin with the V-label, and a third that displays the EU organic certification (BIO) logo. In total, the dataset comprises 10,472 images. Within this collection, the Nutri-Score label is distributed across five sub-labels: Nutri-Score grade A (1,250 images), grade B (1,107 images), grade C (867 images), grade D (1,001 images), and grade E (967 images). Furthermore, there are 870 images showcasing the V-Label, 2,328 images displaying the BIO label, and 3,201 images without any of the aforementioned labels. The methodology presented in this paper holds the potential to extend its utility to address various other food labeling needs. To further enhance the NutriGreen database, the inclusion of supplementary data is possible, provided a sufficient quantity of images featuring branded foods with different labels becomes available.

## Data availability statement

The datasets presented in this study can be found in online repositories. The names of the repository/repositories and accession number(s) can be found at: https://zenodo.org/records/8374047.

## Author contributions

JD: Conceptualization, Data curation, Formal analysis, Methodology, Validation, Visualization, Writing – original draft, Writing – review & editing. IP: Funding acquisition, Writing – review & editing. TE: Conceptualization, Funding acquisition, Investigation, Methodology, Resources, Supervision, Validation, Writing – original draft, Writing – review & editing. BK: Conceptualization, Funding acquisition, Investigation, Project administration, Resources, Supervision, Validation, Writing – review & editing.
